# VHEE FLASH sparing effect measured at CLEAR, CERN with DNA damage of pBR322 plasmid as a biological endpoint

**DOI:** 10.1038/s41598-024-65055-8

**Published:** 2024-06-26

**Authors:** Hannah C. Wanstall, Pierre Korysko, Wilfred Farabolini, Roberto Corsini, Joseph J. Bateman, Vilde Rieker, Abigail Hemming, Nicholas T. Henthorn, Michael J. Merchant, Elham Santina, Amy L. Chadwick, Cameron Robertson, Alexander Malyzhenkov, Roger M. Jones

**Affiliations:** 1 Department of Physics and Astronomy, Faculty of Science and Engineering, University of Manchester, Schuster Building, Oxford Road, Manchester, M13 9PL UK; 2 Manchester Academic Health Science Centre, Christie NHS Foundation Trust, Wilmslow Road, Manchester, M20 4BX UK; 3 Daresbury Laboratory, The Cockcroft Institute, Daresbury, Warrington, WA4 4AD UK; 4 University of Oxford, Oxford, OX1 2JD UK; 5 CERN, Geneva, 1211 Geneva 23, Switzerland; 6 Division of Cancer Sciences, Faculty of Biology, Medicine and Health, School of Medical Sciences, The University of Manchester, Oxford Road, Manchester, M13 9PL UK; 7 University of Oslo, 0316 Oslo, Norway

**Keywords:** Very high-energy electrons (VHEE), FLASH radiotherapy, Relative DNA damage, Hydroxyl radicals, Biological physics, DNA, Cancer

## Abstract

Ultra-high dose rate (UHDR) irradiation has been shown to have a sparing effect on healthy tissue, an effect known as ‘FLASH’. This effect has been studied across several radiation modalities, including photons, protons and clinical energy electrons, however, very little data is available for the effect of FLASH with Very High Energy Electrons (VHEE). pBR322 plasmid DNA was used as a biological model to measure DNA damage in response to Very High Energy Electron (VHEE) irradiation at conventional (0.08 Gy/s), intermediate (96 Gy/s) and ultra-high dose rates (UHDR, (2 × 10^9^ Gy/s) at the CERN Linear Electron Accelerator (CLEAR) user facility. UHDRs were used to determine if the biological FLASH effect could be measured in the plasmid model, within a hydroxyl scavenging environment. Two different concentrations of the hydroxyl radical scavenger Tris were used in the plasmid environment to alter the proportions of indirect damage, and to replicate a cellular scavenging capacity. Indirect damage refers to the interaction of ionising radiation with molecules and species to generate reactive species which can then attack DNA. UHDR irradiated plasmid was shown to have significantly reduced amounts of damage in comparison to conventionally irradiated, where single strand breaks (SSBs) was used as the biological endpoint. This was the case for both hydroxyl scavenging capacities. A reduced electron energy within the VHEE range was also determined to increase the DNA damage to pBR322 plasmid. Results indicate that the pBR322 plasmid model can be successfully used to explore and test the effect of UHDR regimes on DNA damage. This is the first study to report FLASH sparing with VHEE, with induced damage to pBR322 plasmid DNA as the biological endpoint. UHDR irradiated plasmid had reduced amounts of DNA single-strand breaks (SSBs) in comparison with conventional dose rates. The magnitude of the FLASH sparing was a 27% reduction in SSB frequency in a 10 mM Tris environment and a 16% reduction in a 100 mM Tris environment.

## Introduction

The ‘FLASH effect’ is a biological occurrence where healthy tissue is spared post-irradiation. To achieve this effect, high dose rate radiation is used (over ~ 40—100 Gy/s average dose rate^1^) in comparison to conventional dose rates (~ 0.05 Gy/s) that are used clinically. The majority of studies that demonstrate a FLASH effect, as measured by sparing of normal tissues, use in vivo models in multiple species, including mouse, cat and mini-pig.^2–4^. Across these species, healthy tissue sparing has been measured in a range of tissue types including lung^3,5^, brain^1,6–8^, skin^2^, and intestine^9–11^. Several studies have also shown comparable tumour control between conventional and UHDR irradiations, suggesting that UHDRs would be highly beneficial for cancer treatment. In the majority of these studies, electron energies of 4.5 and 6 MeV have been used. Several research groups are currently studying the effects of FLASH using conventional linear accelerators to achieve electrons at UHDR^1,3,12,13^. Many of these studies have taken place at Lausanne University Hospital, where there is a modified electron linac (Oriatron eRT6) available, capable of achieving UHDRs. The Oriatron eRT6 linac was used for the first successful human patient irradiation with UHDR radiotherapy where a 75 year old patient with a resistant lymphoma was prescribed a 15 Gy treatment dose to the skin^14^. The FLASH effect has also been studied with proton and photon radiation and is thought to be independent of modality^15^.

Although the majority of published in vivo studies have shown a biological FLASH effect, there are several studies that have not identified this effect. Wilson et al.^15^ published a systematic review of FLASH studies in 2020 which showed that 3 out of 19 studies did not show significant sparing of healthy tissue. This variation in outcomes shows the importance of investigating the parameters required for the FLASH effect to occur.

The majority of studies use electron radiotherapy, however the effect has also been observed with X-rays and protons, suggesting that this biological effect may be considered modality independent. In 2020, the first human clinical trial for FLASH radiotherapy (FAST-01) opened to enrolment, with patients treated with UHDR protons in a palliative regime for bone metastases^16^. The study has since reported that the delivery of UHDR did not result in any technical issues or delays and that 66.7% of patients treated reported a reduction in pain after FLASH radiotherapy^17^. These initial results have paved the way for the FAST-02 trial^18^, which will focus on thorax bone metastases for palliative patients.

Plasmid DNA are circular DNA molecules that are propagated in bacteria. Plasmids are inert molecules that can be used as biological models to explore the DNA damage effect of radiation without taking into consideration DNA repair or more complex cellular pathways or responses. The environment of the plasmid DNA is easily adapted to alter the amounts of direct and indirect damage that occurs, by the use of chemical scavengers. An insight into the effect of UHDR radiation on DNA damage provides a fundamental first step in determining whether the FLASH effect is due to decreased DNA damage, or a more complex mechanism based on biological pathways.

Many hypotheses exist as to the mechanism underlying the biological FLASH effect however scientists have not yet reached a conclusion as to which mechanism plays the largest part. Hypotheses include oxygen depletion, free radical recombination, inflammation response^19^, circulating immune cells and DNA damage^20^. The oxygen depletion response refers to the fact that areas with available oxygen, such as healthy tissue, are more radiosensitive. When oxygen depletion occurs quickly as with UHDR, the oxygen concentration in healthy tissue is reduced, making the tissue more radioresistant. The free radical recombination hypothesis is linked to this theory; it suggests that irradiation with UHDR causes a sudden increase in reactive oxygen species (ROS)^21,22^. ROS at higher concentrations could result in increased chance of chemical reactions between ROS to form stable molecules, reducing the chance of ROS reacting with DNA. This hypothesis in particular is supported by simulation work^23,24^ which indicates an increase in molecules H_2_ and H_2_O_2_ with higher dose rates (and subsequent reduction of electrons and hydroxyl radicals).

Other hypothesis include a reduced pro-inflammatory response in response to UHDR although no specific mechanism for this has been elucidated as of yet^20^. One hypothesis is specifically focused on the possibility that UHDRs can reduce the proportion of circulating immune cells irradiated^25^, therefore reducing the number of immune cells active in post-irradiation repair. Evidence for the FLASH effect has been observed in vitro across several studies^26^, indicating that the immune cell hypothesis cannot fully explain the FLASH effect alone. In vivo studies have shown biological effects that have challenged mechanistic discovery, are not yet explained or cannot be measured accurately which is why the focus of this study is on the mechanistic discovery of a simpler biological system.

Recently, studies have used plasmid DNA to try and elucidate the mechanism behind the FLASH effect. So far, the FLASH effect has mainly been observed for in vivo models, however it is difficult to pin down exactly what causes the FLASH effect due to the complexity of these models (effects from oxygen concentration, immune response, circulating immune cells, DNA damage, or an unknown mechanism could play a role). Several in vitro studies have tried to measure the FLASH response, however this has proved unsuccessful in the majority of studies^26^. Differences between in vitro and in vivo models (such as oxygen concentration) are thought to be responsible. This study uses a simple plasmid model, with no repair mechanisms, to focus only on any potential differences in DNA damage in response to UHDR.

We reviewed three published studies using plasmids to measure DNA damage in response to UHDRs. The results vary, with two studies reporting a reduction in DNA damage in response to UHDR compared to conventional^27,28^, however one other study reported that DNA damage was independent of dose rate^29^. Our study uses plasmid DNA in two concentrations of hydroxyl scavenging chemical Tris, to determine whether the amount of indirect DNA damage has any effect between dose rates.

Whilst most studies on FLASH use electrons under 10 MeV, Very High Energy Electrons (VHEE) of energies 150 and 201 MeV were the radiation modality used for this experiment. VHEE has been proposed as a potential novel radiotherapy type and importantly, a promising candidate for UHDR radiotherapy. The physical properties of electrons mean they are relatively light (in comparison to protons and heavy ions) and can therefore be delivered and steered efficiently. Higher electron energy translates to increased penetration and decreased spreading of the penumbra^30^, indicating that electrons over ~ 100 MeV could be used to treat deep-seated tumours.

Investigative studies into the biological effect of VHEEs are limited, due in part to their novelty, with only four VHEE accelerators available internationally for users at the current time^31,32^. These include the CERN Linear Electron Accelerator for Research (CLEAR), the Accelerator Research Experiment at SINBAD (ARES), Sources for Plasma Accelerators and Radiation Compton with Lasers and Beams (SPARC) and the Next Linear Collider Test Accelerator (NLCTA). As the field progresses, several more accelerators are in the process of being upgraded to VHEE beam energies^31,33–35^. 150 and 201 MeV electrons were achievable and repeatable using the CLEAR facility. It was important to explore energies within the range of VHEE to add to the very limited body of research with electrons over ~ 35 MeV. In regards to the VHEE energies used in this study, the linear energy transfer (LET) is ~ 0.6–0.7 keV/μm for electrons in the 150–201 MeV range^36^. This is slightly higher than clinically used energies (~ 0.2 keV/μm for 10 MeV electrons). Results from other studies indicate that VHEEs have a relative biological effectiveness (RBE) of approximately 1, or higher, with comparison to a photon reference. Small et al.^29^ used a plasmid model to define VHEE RBE as ~ 1.1–1.2 for electrons in the 100–200 MeV range. Simulation studies have calculated VHEE RBE as 0.99–1.03^37,38^.

Irradiations for this study took place at the CERN Linear Electron Accelerator for Research (CLEAR). The CLEAR electron linac offers users beam energies of 60–220 MeV^39^ and flexible beam parameters that result in a unique ability to alter dose rate regimes from conventional to several orders of magnitude above the FLASH ‘threshold’ of ~ 100 Gy/s^1^. Recent developments to the CLEAR infrastructure, including a robot to handle samples, meant that the plasmids were irradiated with high efficiency and precision^39^. The ability to irradiate up to 32 plasmid samples per batch meant that two concentrations of hydroxyl scavenger Tris could be tested, over a dose range of ~ 15–200 Gy. The unique parameters at CLEAR allowed us to investigate the response of plasmid DNA to different dose rate regimes, at two VHEE energies.

## Materials and methods

### pBR322 plasmid samples

All samples were prepared to total volume of 8 μl within a 0.5 ml Eppendorf tube (*Eppendorf, 0030121023*), containing 100 μg/ml pBR322 plasmid DNA (*New England Biosciences, N3033*) and either 10 or 100 mM Tris hydroxyl scavenger (*Thermo Fisher Scientific, AN9855G*). Distilled water was used to dilute the sample to its final concentration of pBR322 and Tris. The stock plasmid from New England Biosciences contains a residual concentration of Tris–HCl which was included in the calculation of the final concentration of Tris hydroxyl scavenger. The stock plasmid also contains residual EDTA which acts to prevent the degradation of DNA by the deactivation of DNase^40^. 10 and 100 mM concentrations of Tris represent hydroxyl scavenging capacities that are approximately 4% and 40% of a cellular hydroxyl scavenging capacity respectively. This is based on the hydroxyl scavenging capacity of pure Tris (1.1 × 10^9^/M s), which can be used to calculate hydroxyl scavenging capacities of 1.1 × 10^7^/s and 1.1 × 10^8^/s for 10 mM and 100 mM Tris environments respectively^41–43^. Comparisons can then be made with cellular hydroxyl scavenging capacity, taken as 3.0 × 10^8^/s^43^.

### Irradiation of samples at CLEAR, CERN

All samples were irradiated at the CLEAR facility. Samples were processed using gel electrophoresis at the Oglesby Cancer Research Building (OCRB) located in Manchester, UK because facilities to process the plasmid DNA with gel electrophoresis were not available at CERN. Plasmid samples were transported to and from the OCRB on ice and were stored at − 20 °C at all times other than during sample preparation, transport and irradiation. All samples were irradiated with the plasmid solution at equilibrium at room temperature and air conditions (20% oxygen).

**Figure 1:**
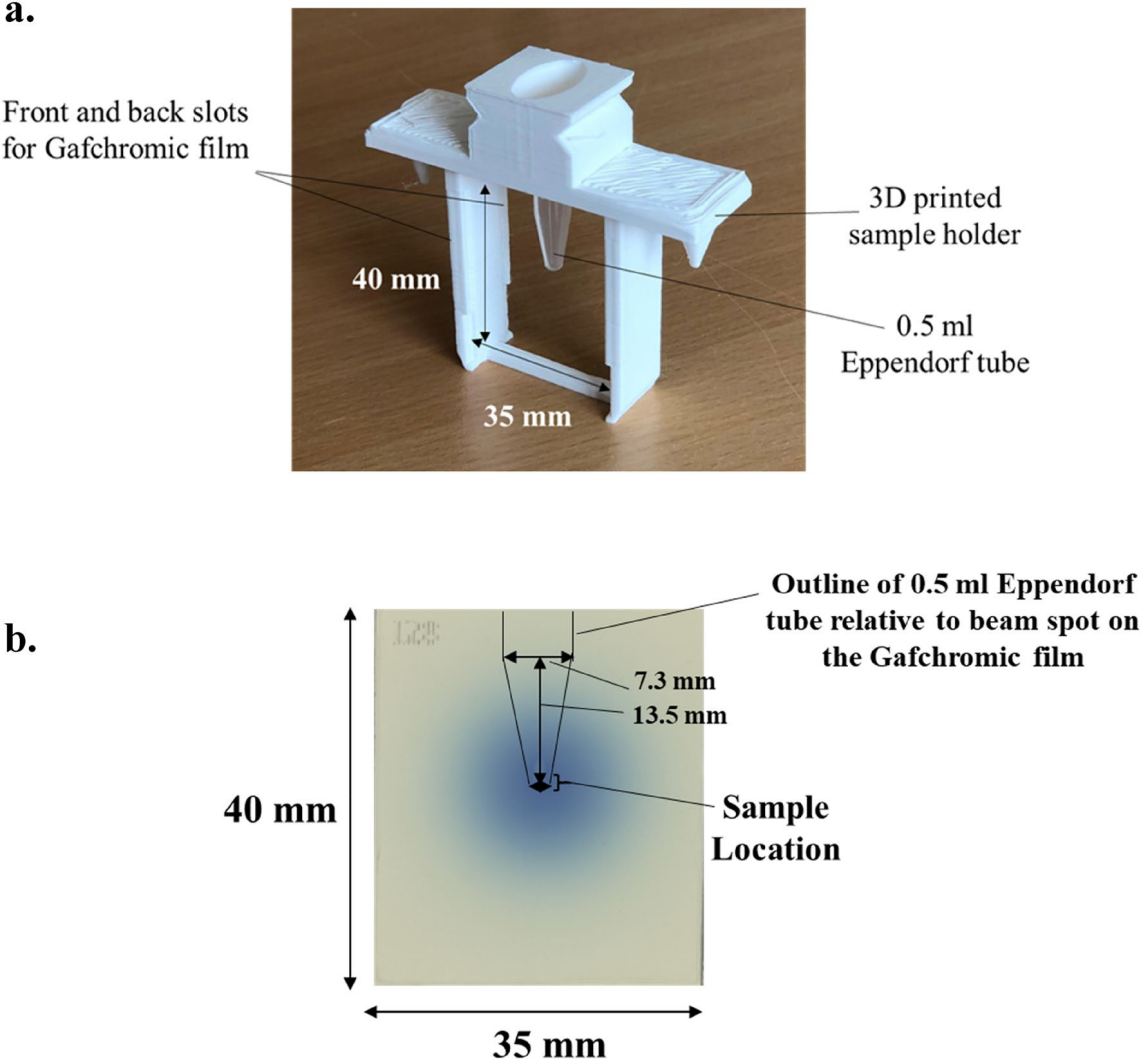
(**a**) Image of the 3D printed sample holders used during the irradiations. 0.5 ml Eppendorf tubes were placed through the entry at the top of the sample holder to secure the tube. Gafchromic films (40 × 35 mm) were then slotted in front and behind each sample to validate dosimetry measurements. (**b**) Scanned Gafchromic film image with reference to the Eppendorf tube and beam spot (represented by the darkening on the film).

Samples were presented to the beam for irradiation using the C-Robot, a device that was used to reduce the turnover time of sample irradiation and number of accesses to the accelerator hall. The C-Robot was controlled by the operator, allowing individual samples to be ‘grabbed’ with high precision and held in front of the beam during the irradiation time. Custom sample holders (Fig. 1) were designed and 3D printed by the CLEAR team to fit the 0.5 ml Eppendorf tubes containing the sample volume.

The samples holders consisted of a round slot in which to place the Eppendorf tube, a ‘handle’ for the C-Robot to grab onto, and two slits for Gafchromic film to be placed in front and behind each sample. EBT-XD Gafchromic film^44^ was used for doses up to 60 Gy. MD-V3 Gafchromic film^45^ was used for samples irradiated in the 60–200 Gy dose range. Up to 32 samples were irradiated in each batch, minimising the frequency of access to the accelerator hall required.

**Figure 2:**
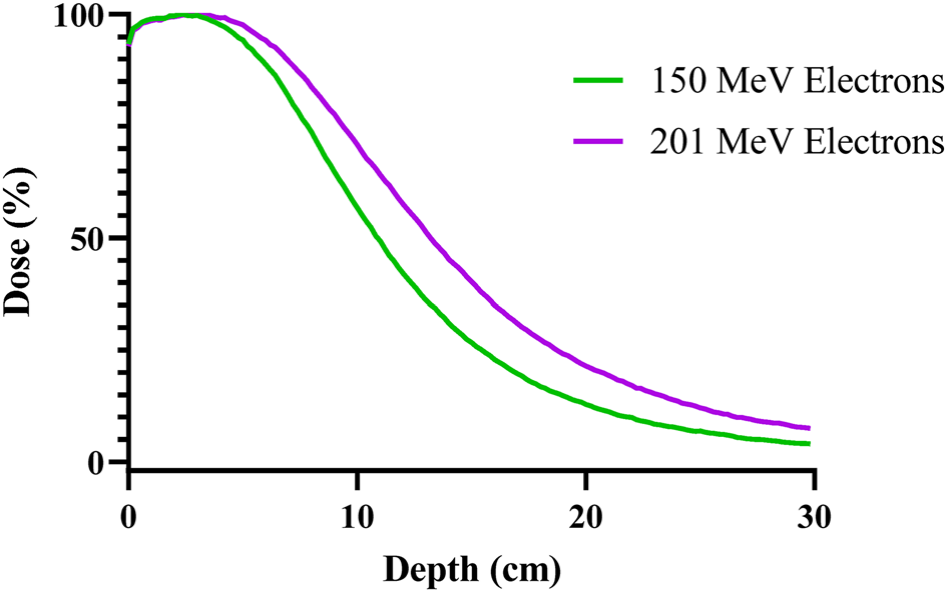
The percentage on-axis dose deposited in respect to depth within a 30 × 30 × 30 cm water phantom for 150 and 201 MeV electrons. Simulation was completed in TOPAS using 10^6^ electrons for each curve, with a Gaussian beam source of σ = 3 mm. Dose was measured across 150 Z axis bins (2 mm × 2 mm × 2 mm each).

Samples were moved to varying depths (1–10 cm) within the water tank to alter beam size. Electron range in water is indicated below in Fig. 2 for context, to show the penetration depth of 150 and 201 MeV electrons using a percentage dose depth curve.

**Figure 3:**
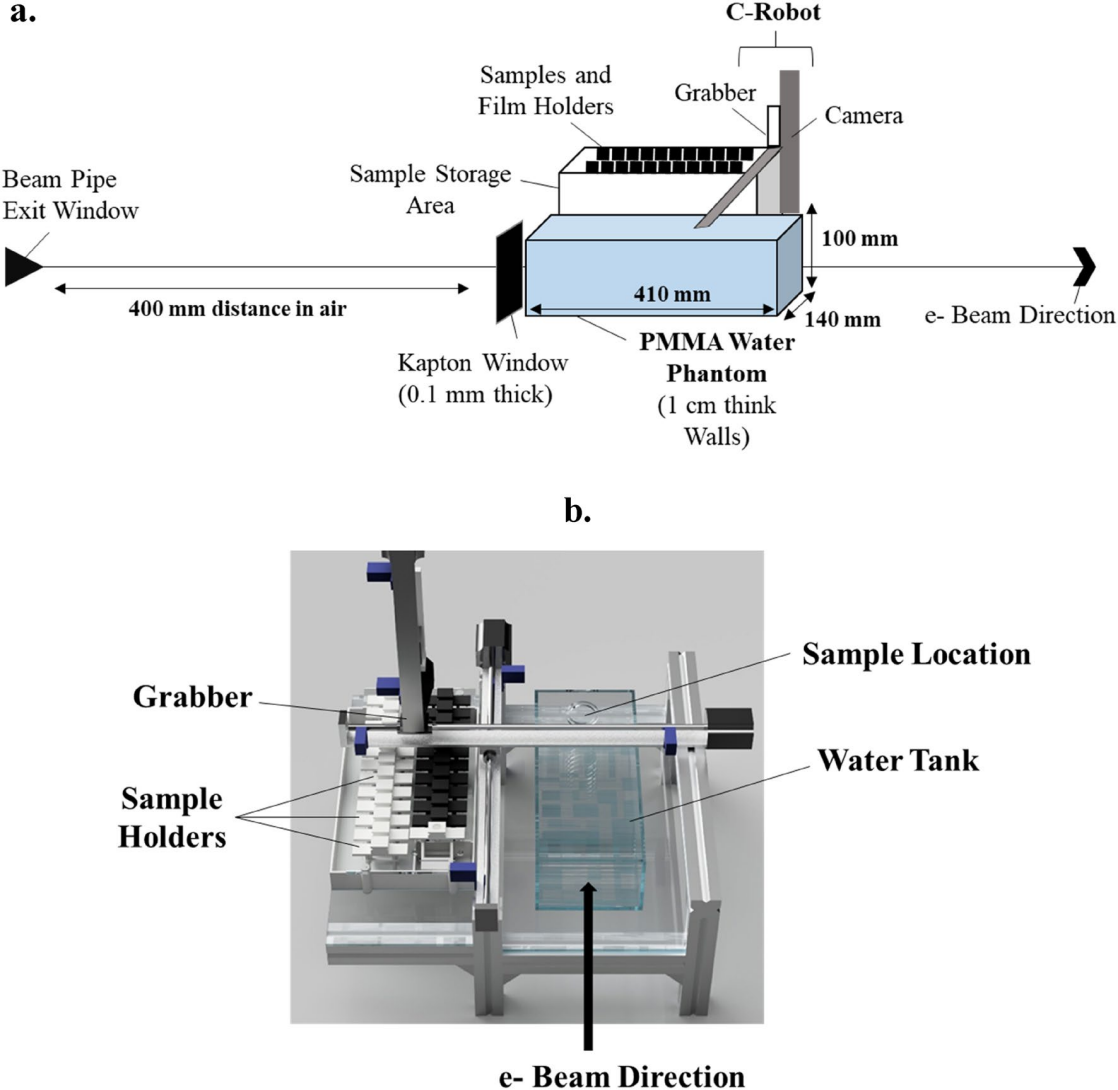
(**a**) Schematic showing the experimental area of the VHEE irradiations at CLEAR. Electrons exited the beam pipe before moving through a 400 mm distance in air. Electrons then passed through a 0.1 mm thick Kapton window and into a water phantom (410 × 140 × 100 mm as labelled in this figure). The water phantom was made of PMMA with the phantom walls having a consistent thickness of 1 cm. Samples and film holders were stored in the sample storage area throughout the experiment. To irradiate samples, the grabber would retrieve the samples by grabbing its 3D printed holder and placing it at a depth of between 10 and 100 mm depth into the water phantom for the irradiation to take place. Samples would then be returned to the sample storage area. (**b**) 3D image of the experimental area.

The beam in air before entry to the water tank was a symmetrical Gaussian beam, σ = 0.8–1.2 mm. The beam at the sample depth consisted of a symmetrical Gaussian beam, σ = 2.8–5.5 mm. In regards to electron fluence, a 5.5 × 5.5 mm σ beam size required ~ 1 nC to achieve 1 Gy. For a 5.0 × 5.0 mm σ beam size, ~ 0.8 nC was required to achieve 1 Gy. Experimental set-up is indicated below in Fig. 3.

### Calculation of sample dose and dose rate

Dose was measured from either EBT XD or MDV3 Gafchromic film by analysing within the irradiated sample area. The sample area was located on the film images by using example films which were placed behind a 0.5 ml Eppendorf tube containing a small lead pellet, which was then irradiated. The lead pellet absorbed a significant amount of dose so that a visible spot could be located on the film. This spot was then used to determine where the bottom of the Eppendorf tube, and therefore the sample was located. Once the location of the sample area was isolated, the mean dose was measured from the film placed directly behind and in front of each sample. Average dose rate was calculated for conventional, intermediate and ultra-high dose rate (UHDR) by measuring the dose to sample with Gafchromic film and dividing by the total irradiation time. Average dose rates were calculated as follows: 0.08 Gy/s (conventional), 96 Gy/s (intermediate) and of the order of 2 × 10^9^ Gy/s (UHDR). Physical parameters are defined for each dose rate used below in Table 1.

**Table 1:** Physical parameters of the CLEAR beamline for each dose rate used to irradiate plasmid samples.

	UHDR	Intermediate	Conventional
Bunch charge (pC)	1000	70	200
Bunch length (ps)	2.5	2.5	2.5
Bunch spacing (ps)	666	666	666
Bunches per train	Variable	100	1
Repetition rate (train/s)	N/A – irradiation in one train	10	0.8
Average dose rate (Gy/s)	2 × 10^9^	96	0.08
Dose rate (in bunch) (Gy/s)	3 × 10^11^	4 × 10^10^	4 × 10^10^
Dose rate (in train) (Gy/s)	2 × 10^9^	10^8^	4 × 10^10^

### Separation of plasmid structures with gel electrophoresis

Irradiation of plasmid DNA within the dose range used for this experiment results in damage in the form of single- and double- strand DNA breaks, known as SSBs and DSBs respectively. The native structure of pBR322 plasmid is supercoiled (SC). Plasmids that have been damaged by a SSB or DSB will subsequently change structure. Plasmids with damage in the form of an SSB will display an open circular (OC) structure, whereas a more damaging DSB will result in a linear (L) structure. SC, OC and L structures can be therefore be separated by gel electrophoresis due to their subsequent differences in plasmid mobility.

All plasmid samples were separated using gel electrophoresis methods. This was completed at the Oglesby Cancer Research Building (OCRB), UK. 1% w/v agarose gel was prepared with 1× TAE buffer (*Thermo Fisher Scientific, B49*), agarose powder (*Sigma Aldrich, A9539*) and 1× SYBR Safe DNA gel stain (*Thermo Fisher Scientific, S33102*). To process the samples with gel electrophoresis, 83.3 μg/ml pBR322 plasmid DNA and 1X loading dye (*Thermo Fisher Scientific, R0611*) is loaded into the gel at a total volume of 1 μl. Once set, the gel was placed in an electrophoresis tank and covered in 0.5× TAE buffer (*Thermo Fisher Scientific, B49*). A constant voltage of 70 V was then applied for 2.5 h.

Gels were imaged on the ChemiDoc MP UV imager (BioRad) using the ‘SYBR Safe’ setting, with the exposure condition always set to ‘Automatic Rapid Exposure’.

### Quantification of DNA damage with the McMahon fit

To quantify the relative amount of plasmid DNA in each proportion, the integrated density of each band within the electrophoresis gel was measured using Image J software. The number of induced SSBs and DSBs was then calculated using the McMahon and Currell fitting procedure^46^. The curves were fit to data points using Microsoft Excel Solver (2016) by calculating curves that minimised the least squared fit. Values of β_*s*_ and β_*D*_ (SSB and DSB respectively) were calculated by minimised the square of the error for the measured SC, OC and L proportions and the McMahon and Currell fit.

1 $$SC = SC_{0} e^{{ - (\beta_{s} + \beta_{D} )D}}$$

2 $$OC = e^{{ - \beta_{D} D}} \left[ {OC_{0} e^{{ - \frac{1}{2}\beta_{s}^{2} \rho D^{2} }} + SC_{0} \left( {e^{{ - \frac{1}{2}\beta_{s}^{2} \rho D^{2} }} - e^{{ - \beta_{s} D}} } \right)} \right]$$

3 $$L = 1 - (OC_{0} + SC_{0} )e^{{ - \left( {\beta_{D} D + \frac{1}{2}\beta_{s}^{2} \rho D^{2} } \right)}}$$

where *SC*, *OC* and *L* are the proportions of supercoiled, open circular and linear plasmid structures respectively. *SC*_*0*_, *OC*_*0*_ and *L*_*0*_ are the mean proportions of supercoiled, open circular and linear plasmid structures in unirradiated controls for 10 and 100 mM Tris concentrations as applicable. *D* represents the dose to sample in Gy. *ρ* represents the ratio between the length of pBR322 plasmid (4361 bp) and the maximum distance between two SSBs resulting in a DSB (taken as 10 bp^29,47^). β_*s*_ and β_*D*_ are the frequencies of SSBs and DSBs per unit dose across the range of doses used to irradiate the plasmid samples.

Samples were irradiated in various conditions during the experiment, with several doses for each condition. In the majority of cases, enough dose points were used so that a McMahon fit could be made and SSB and DSB frequencies could be calculated with a degree of statistical rigour. In the case of the intermediate dose rate irradiations, irradiations were only successfully completed at two doses and therefore the McMahon curve was not utilised to calculate SSB or DSB frequencies for this data. This was because the limited number of doses was not deemed to be as statistically rigorous as other conditions with a larger number of dose points. The intermediate data at two doses is plotted in Fig. 5, however a McMahon fit has not been plotted alongside for this reason.

### Statistical analysis

Values of SSB induction for each condition were calculated using McMahon fits as described above. The 95% and 68% confidence intervals (CI) were calculated on each SC and OC McMahon fit using a custom Python code. The weighted 95% CI that considered both SC and OC fits is presented in Table 2 and is plotted as the error values in Figs. 8 and 9.

**Table 2:** Values of SSB (β_S_) and DSB (β_D_) induction as predicted by the McMahon model for each experimental condition.

Electron energy (MeV)	[Tris]	Dose rate	SSB (/Mbp Gy)		DSB (/Mbp Gy)	
			Mean	95% CI on McMahon fit	Mean	95% CI on McMahon fit
201	10 mM	CONV	3.05	± 0.08	0.06	± 0.03
		UHDR	2.24	± 0.14	0.05	± 0.07
	100 mM	CONV	0.68	± 0.07	0.04	± 0.04
		UHDR	0.57	± 0.21	0.04	± 0.10
150	10 mM	CONV	3.40	± 0.17	0.11	± 0.07

Error values represent the weighted 95% confidence interval (CI) of the McMahon fits to the corresponding SC and OC curves presented in Figs. 5, 6 and 7.

To determine statistical significance, an Unpaired t-test was completed, where a *P*-value of < 0.05 was considered significant. For the t-test, the 68% CI on either the SC or OC McMahon curve was taken as the standard deviation, whichever was the more significant error. *N* was taken as the number of irradiated samples for each curve where *N* = 8–12.

## Results

**Figure 4:**
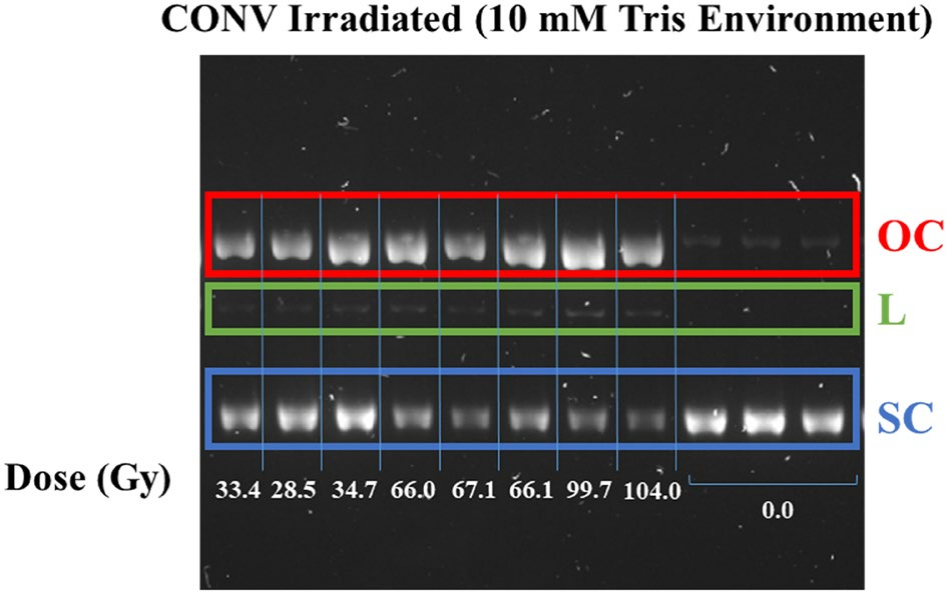
Image of agarose gel of pBR322 plasmid samples irradiated with conventional (CONV) 201 MeV electrons, in a 10 mM environment. Plasmid DNA is separated out into three structures: Open circular (OC)—top band, red. Linear (L), middle band, green. Supercoiled (SC)—bottom band, blue. The image represents the original image as taken with no processing. Dose (Gy) is indicated for each band on the gel by the labelling on the bottom of the figure. Unirradiated samples are indicated by the three lanes labelled 0.0 Gy. Each of the displayed samples were derived from the same experiment and gels were processed in parallel. Gels were imaged on the ChemiDoc MP UV imager (BioRad) using the ‘SYBR Safe’ setting, with the exposure condition always set to ‘Automatic Rapid Exposure’. This figure is a cropped section of a gel—the full gel image can be seen in the supplementary section.

Plasmid samples were irradiated at various doses (up to ~ 200 Gy). All pBR322 plasmid structures were then separated via gel electrophoresis as previously described. Figure 4 shows an example of how plasmid structure changes in response to dose. Over 150 plasmid samples were processed this way, therefore Fig. 4 shows a small proportion of the samples, as an example only. Figure 4 shows three unirradiated samples as well as a selection of samples irradiated with conventional 201 MeV electrons in a 10 mM Tris environment.

It can be seen that for the unirradiated (0 Gy) plasmid samples, the majority of plasmid remains in the native SC structure, where band intensity is visibly strong. Fainter bands can be seen in the OC structure, indicating a smaller percentage of the plasmid is in this structure. No visible band can be seen in the L structure, suggesting no or very few DSBs have occurred in the unirradiated sample. This is verified by measuring the integrated density of each plasmid structure in its unirradiated form. Unirradiated samples within a 10 mM Tris environment were measured to have an average SC proportion of 0.89 ± 0.04 with a corresponding OC proportion of 0.08 ± 0.03, and a smaller L proportion of 0.03 ± 0.03. Errors represent standard deviation. Increasing the concentration of Tris to 100 mM was shown to significantly increase the proportion of plasmid remaining in its native SC structure, protecting the plasmid from base levels of damage during transport and storage. Unirradiated samples maintained within a 100 mM Tris environment had an average SC proportion of 0.98 ± 0.03, an OC proportion of 0.02 ± 0.03 and a corresponding L proportion of 0.001 ± 0.003.

**Figure 5:**
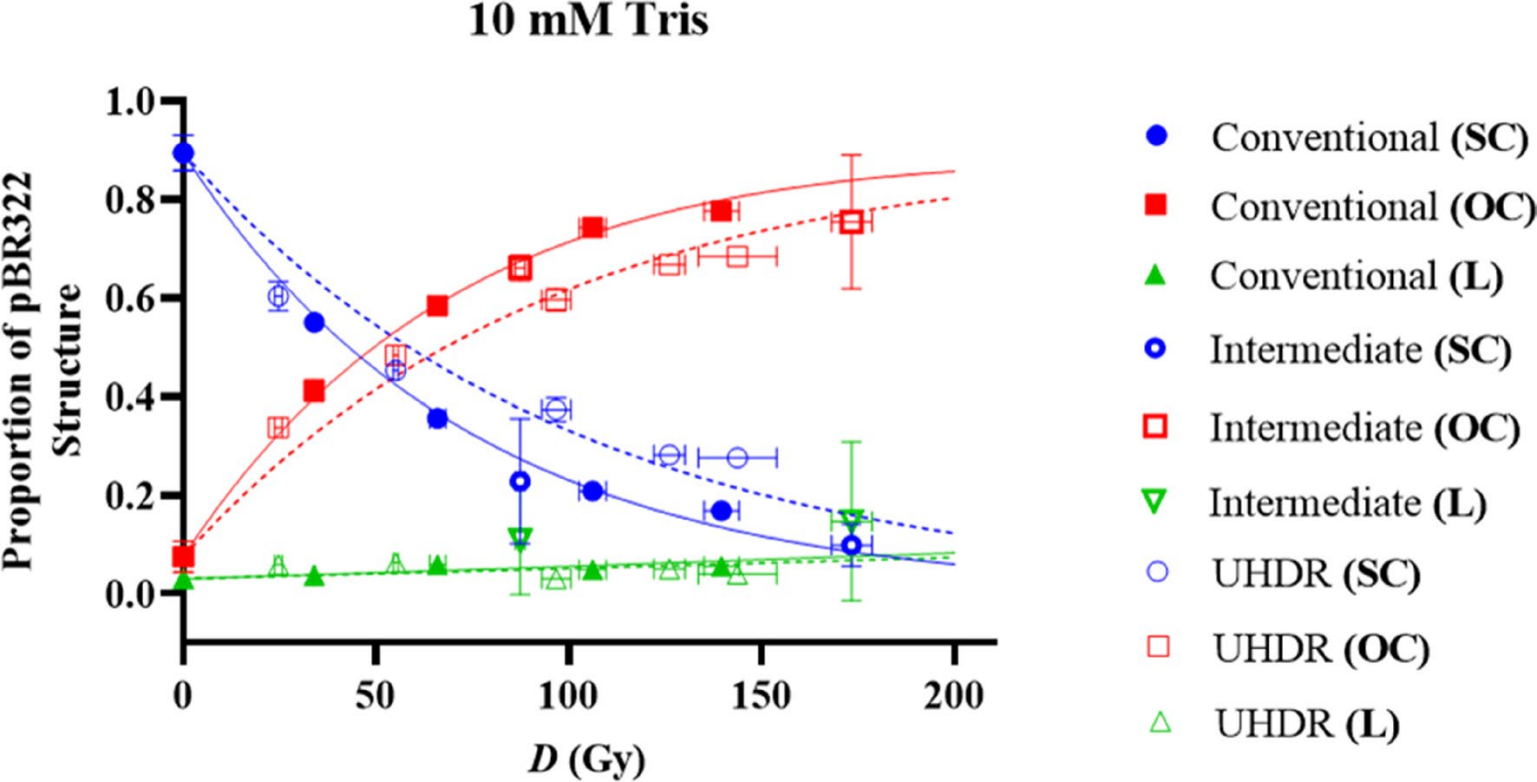
Relative proportions of SC (red), OC (blue) and L plasmid (green) relative to dose (*D*) in Gy within a 10 mM Tris environment. Irradiations were completed with 201 MeV electrons. The effect of dose rate on DNA damage is indicated here, with conventional, intermediate and ultra-high dose rate (UHDR) representing dose rates of 0.08, 96 and in the order of 2 × 10^9^ Gy/s respectively. The solid and dotted lines represent the McMahon fits to the conventional and UHDR data sets respectively. No fit has been added for the intermediate dose rate because the data set only included two doses which was not deemed enough points to make a reliable fit. Points represent the mean dose and plasmid proportions of 2 or 3 irradiation repeats. Error bars represent standard deviation (SD) in both X and Y.

**Figure 6:**
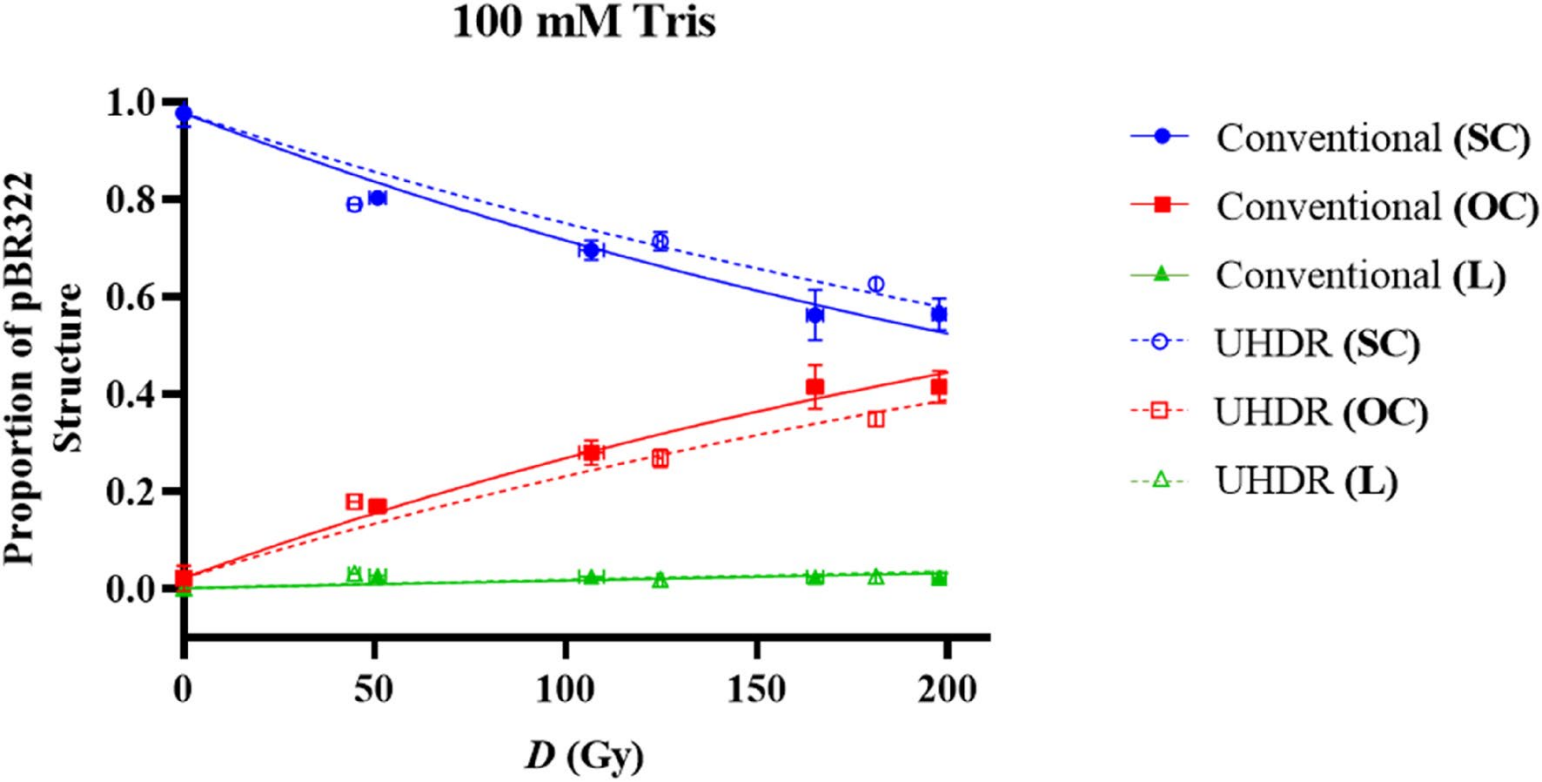
Relative proportions of SC (red), OC (blue) and L plasmid (green) relative to dose (*D*) in Gy within a 100 mM Tris environment. Irradiations were completed with 201 MeV electrons. The effect of dose rate on DNA damage is indicated here, with conventional and ultra-high dose rate (UHDR) representing dose rates of 0.08 and in the order of 2 × 10^9^ Gy/s respectively. The solid and dotted lines represent the McMahon fits to the conventional and UHDR data sets respectively. Points represent the mean dose and plasmid proportions of 2 or 3 irradiation repeats. Error bars represent standard deviation (SD) in both X and Y.

**Figure 7:**
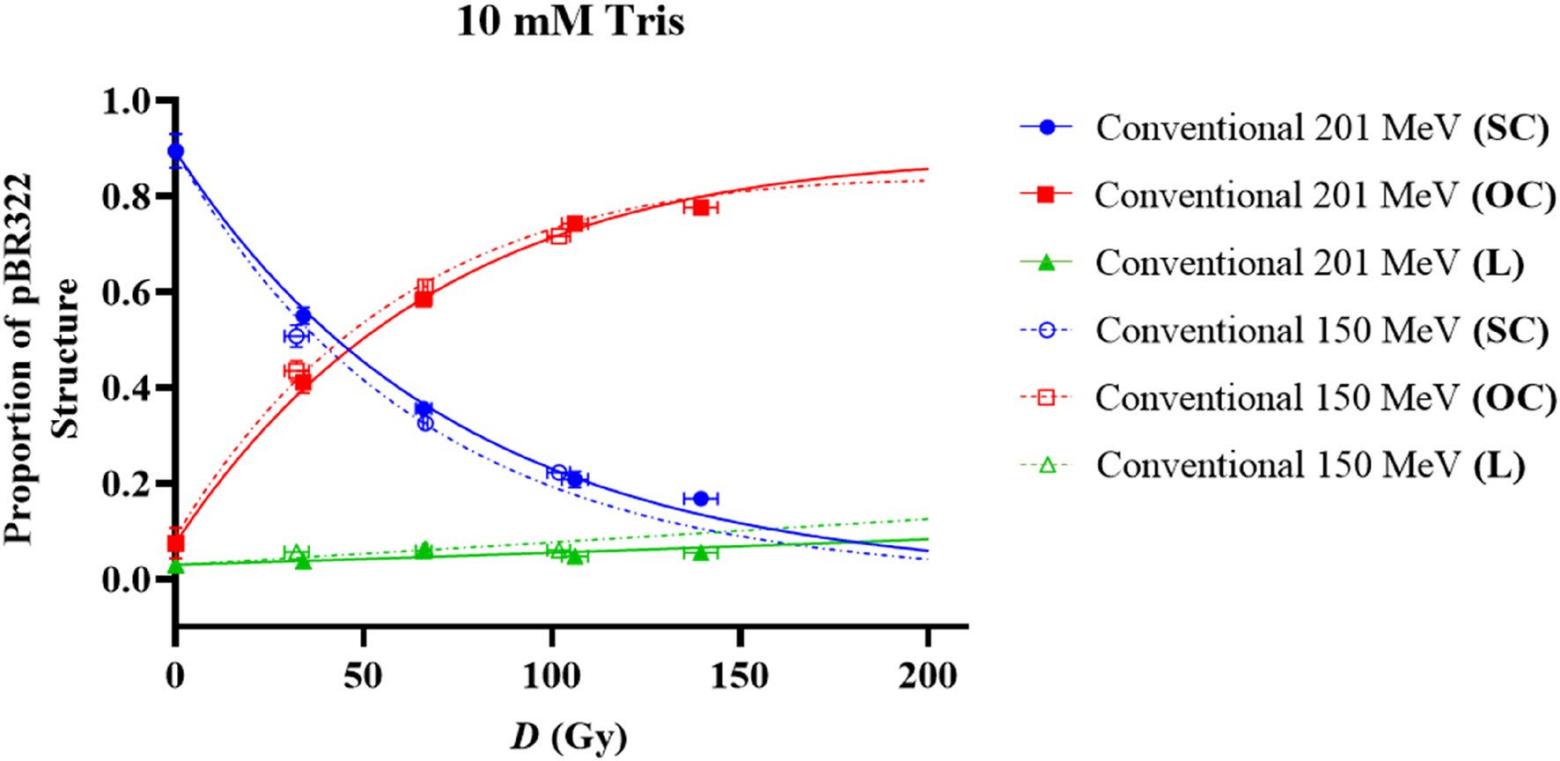
Relative proportions of SC (red), OC (blue) and L plasmid (green) relative to dose (*D*) in Gy within a 10 mM Tris environment. Irradiations were completed with either 150 or 201 MeV electrons at conventional dose rates. The effect of beam energy on DNA damage is indicated here, with solid and dotted lines representing the McMahon fits to the 201 MeV and 150 MeV irradiated sets respectively. Points represent the mean dose and plasmid proportions of 2 or 3 irradiation repeats. Error bars represent standard deviation (SD) in both X and Y.

Figure 4 also shows how the quantity of plasmid structures changes when irradiated with doses in a range of 28.5–104.0 Gy. Post-irradiation, more intense bands can be observed for the OC structure in comparison to unirradiated plasmid indicating a higher frequency of SSBs for irradiated plasmid. Differences cannot be easily distinguished between the proportions of plasmid in OC and L structures at each dose. It can however be identified with visual inspection that the quantity of plasmid in SC structure decreases with increased dose based on the band intensity. This is quantified for each experimental condition in Figs. 5, 6 and 7.

The proportions of pBR322 plasmid structure (SC, OC and L) for every experimental condition have been plotted in response to dose (*D*) in Figs. 5, 6 and 7. Figure 5 shows the response of samples irradiated with 201 MeV electrons, in an environment of 10 mM Tris. The structure proportions of pBR322 for conventional, intermediate and ultra-high dose rate (UHDR) have been plotted to compare between dose rates. McMahon fits for conventional and UHDR conditions have also been included.

Figure 6 shows the response of pBR322 plasmid irradiated with 201 MeV electrons, in a 100 mM environment. pBR322 structure proportions for conventional and UHDRs are indicated as well as the corresponding McMahon fits.

Irradiations were completed at two VHEE energies: 150 and 201 MeV. Figure 7 represents the effect of these two energies on the pBR322 plasmid structure by comparing conventional irradiated samples, in a 10 mM Tris environment.

A summary of SSB (β_S_ in Eqs. 1–3) and DSB (β_D_) frequencies (/Mbp Gy) for each experimental condition is summarised below in Table 2 alongside the confidence in corresponding McMahon fits.

**Figure 8:**
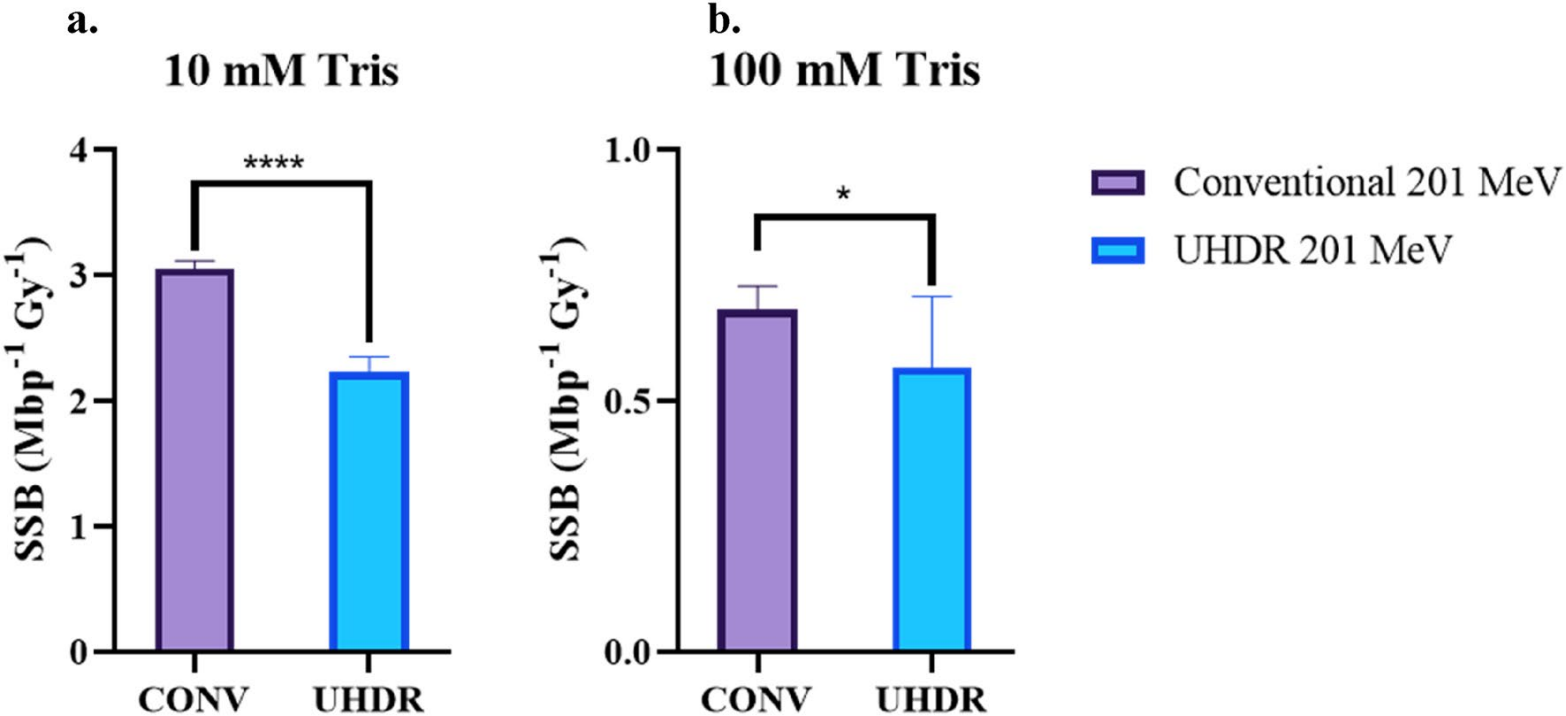
The effect of dose rate on SSB induction is shown here in both a 10 mM Tris environment (**a**) and 100 mM Tris environment (**b**). Conventional and ultra-high dose rate (UHDR) representing dose rates of 0.08 and in the order of 2 × 10^9^ Gy/s respectively. Error bars represent the weighted 95% confidence intervals of the McMahon fits to the corresponding SC and OC curves, as shown in Table 2. **** = statistical difference where *P* ≤ 0.0001, * = statistical difference where 0.01 ≤ *P* ≤ 0.05.

Data summarised in Table 2 has been plotted in Fig. 8 to compare between the DNA damage induced by conventional and UHDR irradiations. SSB frequency is the biological endpoint used to perform the statistical t-test due to the significantly higher frequency of SSBs than DSBs. Figure 8a and b show the SSB induction to conventional and UHDR irradiations in a 10 mM and a 100 mM Tris environment respectively. Based on the response of an unpaired t-test, SSB induction is lower in response to UHDR irradiation than conventional irradiation in both conditions of Tris hydroxyl scavenger.

**Figure 9:**
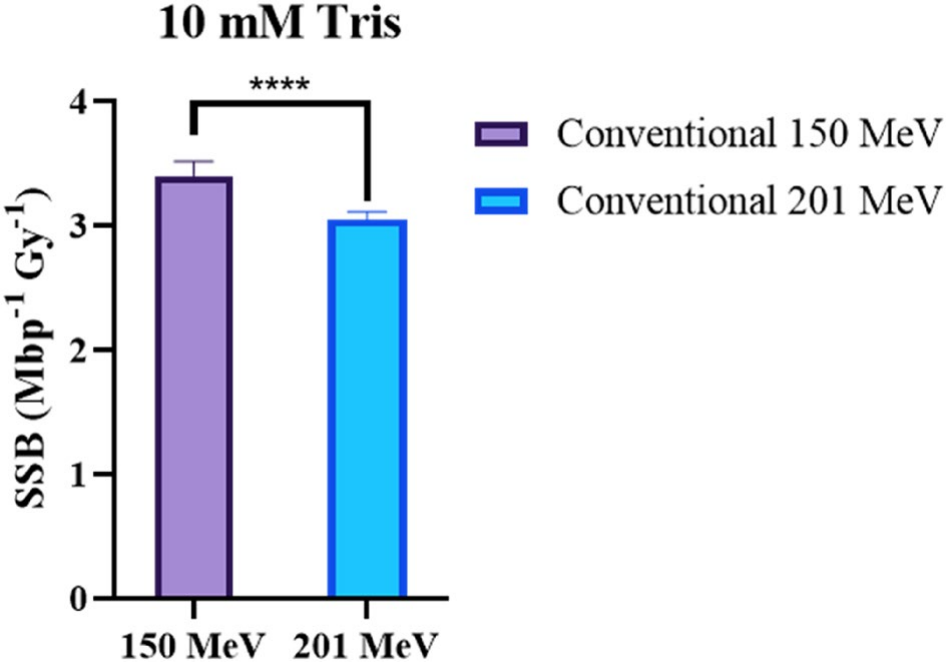
The effect of electron energy on SSB induction is shown here in a 10 mM Tris environment. Irradiations took place at a conventional dose rate with a beam energy of either 150 or 201 MeV. Error bars represent the weighted 95% confidence intervals of the McMahon fits to the corresponding SC and OC curves, as shown in Table 2. **** = statistical difference where *P* ≤ 0.0001.

Figure 9 shows the SSB induction in response to irradiations of two different electron energies: 150 MeV and 201 MeV. Both electron energies were irradiated at conventional dose rates within a 10 mM Tris environment. A t-test indicated that 150 MeV electrons had significantly lower SSB frequency in comparison to 201 MeV electrons.

Proportional values have been calculated from the data shown in Table 2 above, as an indication of the DNA damage inflicted by ultra-high dose rate, and for 150 MeV electron irradiations (in comparison to 201 MeV, conventional dose rate electrons). These proportional values are shown in Table 3.

**Table 3:** Proportional values of SSB frequency calculated from values of SSB (β_S_) induction as provided in Table 2.

Electron energy (MeV)	[Tris]	Dose rate	SSB_INTEREST_/SSB_REFERENCE_ (with 201 MeV CONV irradiated samples as the reference)
201	10 mM	CONV	1.00
		UHDR	0.73
	100 mM	CONV	1.00
		UHDR	0.84
150	10 mM	CONV	1.11

Values were calculated using SSB_INTEREST_/SSB_REFERENCE,_ where SSB_INTEREST_ is the frequency of SSBs (/Mbp Gy) induced by the radiation of interest (UHDR or 150 MeV conventional)^29^. SSB_REF_ describes the frequency of SSBs (/Mbp Gy) induced by the reference radiation type (201 MeV, conventional dose rate irradiation). Only values of the same Tris concentration were used to calculate proportional values, e.g. where values was calculated for UHDR in a 10 mM environment, the reference used would be conventional irradiated samples in a 10 mM environment.

## Discussion

The aim of this study was to investigate the biological effect of dose rate and beam energy in the context of VHEE. The results show that UHDR irradiation significantly reduces SSB induction in comparison to conventional irradiation. This is the case in two hydroxyl scavenging environments, 10 mM and 100 mM Tris. Increased concentration of Tris scavenger resulted in a significant reduction in damage to the plasmid DNA due to the reduction of hydroxyl- mediated indirect DNA damage. The results also indicated that the SSB induction was significantly increased in plasmid samples irradiated with 150 MeV electrons, in comparison to plasmid irradiated at 201 MeV.

In a 10 mM Tris environment, pBR322 plasmid irradiated with 201 MeV electrons resulted in a SSB frequency of 3.05 ± 0.08 (conventional) and 2.24 ± 0.14 (UHDR) (/Mbp Gy). Corresponding DSB frequencies of 0.06 ± 0.03 (conventional) and 0.05 ± 0.07 (UHDR) (/Mbp Gy) were also observed. At 10 mM Tris, a set of irradiations were also completed using a 150 MeV electron beam. At 150 MeV, the SSB and DSB frequencies were 3.40 ± 0.17 and 0.11 ± 0.07 (/Mbp Gy) respectively for conventional irradiated plasmid.

In the 100 Mm Tris environment, the frequency of SSBs was reduced significantly. In the 100 mM environment with 201 MeV electron irradiation, the SSB frequency 0.68 ± 0.07 (conventional) and 0.57 ± 0.21 (UHDR) and the DSB frequency was 0.04 ± 0.04 (conventional) and 0.04 ± 0.10. Due to the high error on the DSB frequencies, that data has not been plotted and the endpoint of SSB frequency has been used to compare between dose rates and beam energies.

This is the first time that FLASH sparing has been reported using VHEE, with induced damage to pBR322 plasmid DNA as the biological endpoint. The results of this study are in contrast to those published by Small et al.^29^ in 2021, where plasmid samples were also irradiated at the CLEAR user facility, at conventional and UHDR. There are some minor differences in the methodology between this study and those published by Small et al., the main difference being that this study used a Tris concentration in a 10–100 mM range whereas Small et al., used a 1 mM Tris concentration throughout. Another key difference is that Small et al., used DSB frequency as the biological endpoint whereas for this study, it was determined that the statistical frequency of DSBs was not large enough to determine significant differences between dose rates. Therefore DSBs were not used to make these comparisons.

On the other hand, the results of this study are supported by two other studies that have observed DNA damage sparing to plasmid in response to 27.5 MeV protons^27^, and 16 MeV electrons^28^. Ohsawa et al., reported that UHDR (40 Gy/s) protons resulted in sparing of pBR322 plasmid damage where SSB frequency was the biological endpoint measured. The plasmids were irradiated in an environment containing 10 mM Tris–HCl. Similar to this study, Ohsawa et al., found that UHDR irradiation reduced DSB frequency however this was not to a statistically significant level.

This could indicate that the biological FLASH effect does not translate to DSBs, suggesting that the most lethal form of damage is still maintained, with a reduction in only sub-lethal damages. Another interpretation is that the significantly lower frequency of DSBs in comparison to SSBs means that it is not statistically viable to determine a difference using DSB frequency as the biological endpoint using published methods. To fully understand the effect of dose rate on DSB induction, larger volumes of plasmid, higher doses or the removal/reduction of hydroxyl scavengers from the plasmid environment could be used to increase DSB frequency. These alterations to the protocol could be tested in further studies, but these changes could be limited by cost, beam time duration or dosimetry, if using doses out of the range of Gafchromic film. Overall, the study by Ohsawa et al., indicates that the FLASH effect takes place in a plasmid model, and that this is independent of modality, as has also been shown in vivo.

Perstin et al., tested the effect of 16 MeV electrons at conventional and UHDR (46.6 and 93.2 Gy/s) in an environment of 0.48 mM Tris–HCl. It was determined from this study that the FLASH sparing effect was significant at both dose rates, with a more pronounced effect for SSB induction than for DSBs, even for this low concentration of Tris scavenger.

Overall, these collective studies indicate that using the pBR322 plasmid model is an effective way to identify factors that impact DNA damage sparing, providing insight into measuring the biological FLASH effect. This information should be used to inform more complex experiments such as in vitro and in vivo work to elucidate a biological mechanism for FLASH.

In vitro studies have mixed results when using biological endpoints to measure the FLASH effect. A recent review^26^ investigated the current breadth of research in this area, reporting that 6 out of 19 studies measuring cell survival showed evidence of the FLASH effect in any condition (in hypoxic or normoxic conditions, or in cancer or normal cells). Despite the varying reports of FLASH in vitro, there is evidence of UHDRs resulting in sparing of DNA damage linked biological effects. UHDRs have been shown to reduce dicentric yields in irradiated human blood^48^. Reduced levels of DNA damage markers including γH2AX and 53BP1 have also been observed in both tumour and normal cells with UHDRs, in comparison to conventional dose rates^5,49^. These studies support the hypothesis that one of the major mechanisms for the FLASH effect is by a reduction in DNA damage, however more mechanistic information is required to fully understand the biological FLASH mechanism from irradiation to in vivo impact.

The FLASH effect has now been observed in plasmids within Tris environments from 0.48 to 100 mM, and in response to protons, electrons and VHEE. Plasmid DNA can and should be used to test a wide range of UHDR regimes to get an indication for the necessary parameters for the FLASH effect. These parameters could then be used to guide in vivo studies.

It should be noted however that a measuring SSB frequency alone does not paint a full picture of the biological FLASH effect. The quantity of DNA damage is important, however it must be used as a first step to facilitate further studies on how DNA damage affects FLASH endpoints that occur at later time points, including DNA repair, cell cycle and cell survival.

### Supplementary Information

Supplementary Figure 1.

## Data Availability

The main data in this article is presented in table and graph format throughout. Any other data or specific information required will be shared upon reasonable request to the corresponding author. Authors confirm that no tissue samples or cell lines were used for this study.
