# Supplementary material for: VHEE FLASH sparing effect measured at CLEAR, CERN with DNA damage of pBR322 plasmid as a biological endpoint
Source: Sci Rep. 2024 Jun 26;14:14803. doi: 10.1038/s41598-024-65055-8 (PMC11208499; doi:10.1038/s41598-024-65055-8)
Supplement: Supplementary file 1 — Supplementary Figure 1. [file 41598_2024_65055_MOESM1_ESM.pdf]

Supplementary.

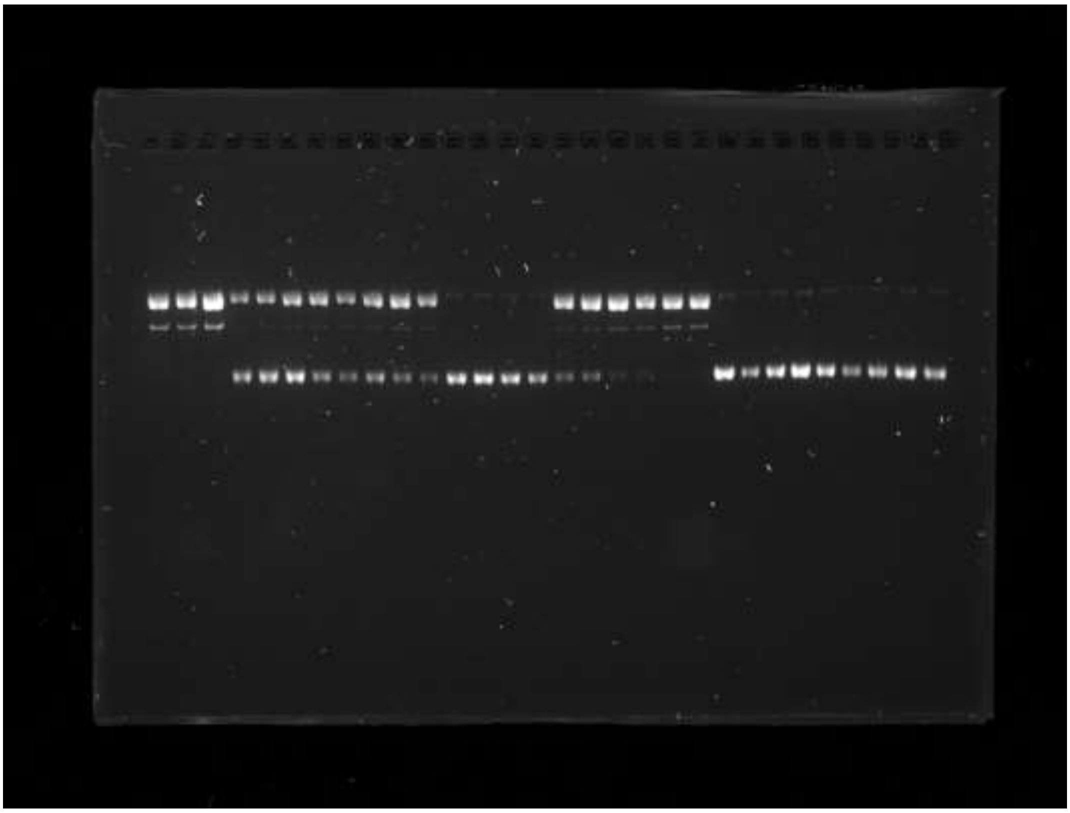

Supplementary Figure 1. Full, uncropped gel electrophoresis image from which Figure 3 was cropped. Figure 3 includes bands 4 – 14 as a representation of how increased dose changes plasmid structure proportion.
